# Supplementary material for: Dihydroconiferyl Ferulate Isolated from Dendropanax morbiferus H.Lév. Suppresses Stemness of Breast Cancer Cells via Nuclear EGFR/c-Myc Signaling
Source: Pharmaceuticals (Basel). 2022 May 26;15(6):664. doi: 10.3390/ph15060664 (PMC9231027; doi:10.3390/ph15060664)
Supplement: Supplementary file 1 [file pharmaceuticals-15-00664-s001.zip › pharmaceuticals-1692781-supplementary.pdf]

Positive mode

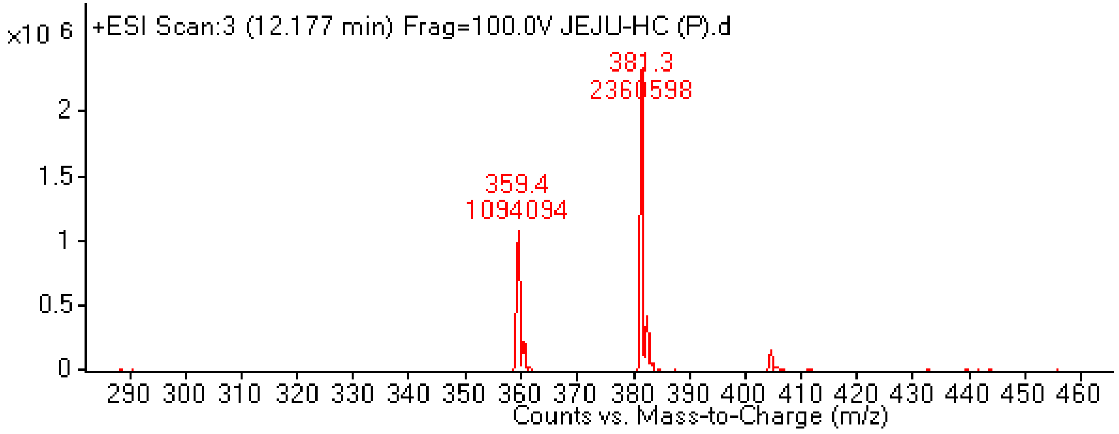

Negative mode

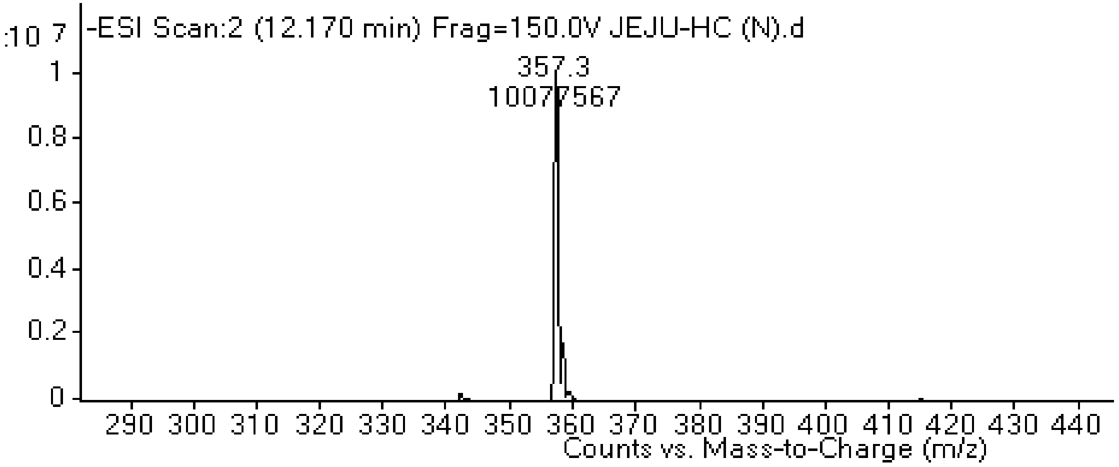

Figure S1. LC-ESI-mass spectrometry of the purified compound.

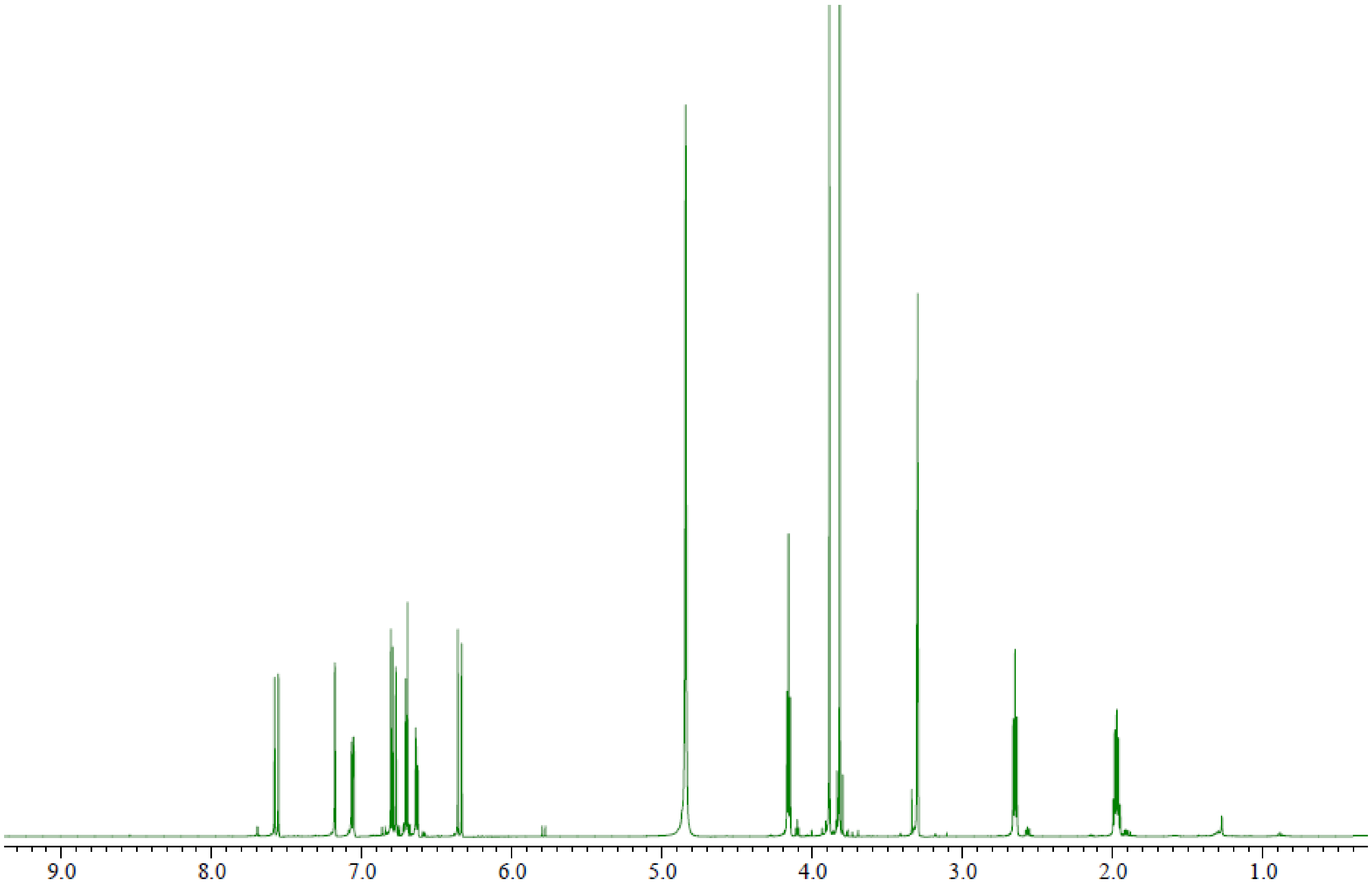

Figure S2. ^{1}H NMR spectrum of the purified compound.

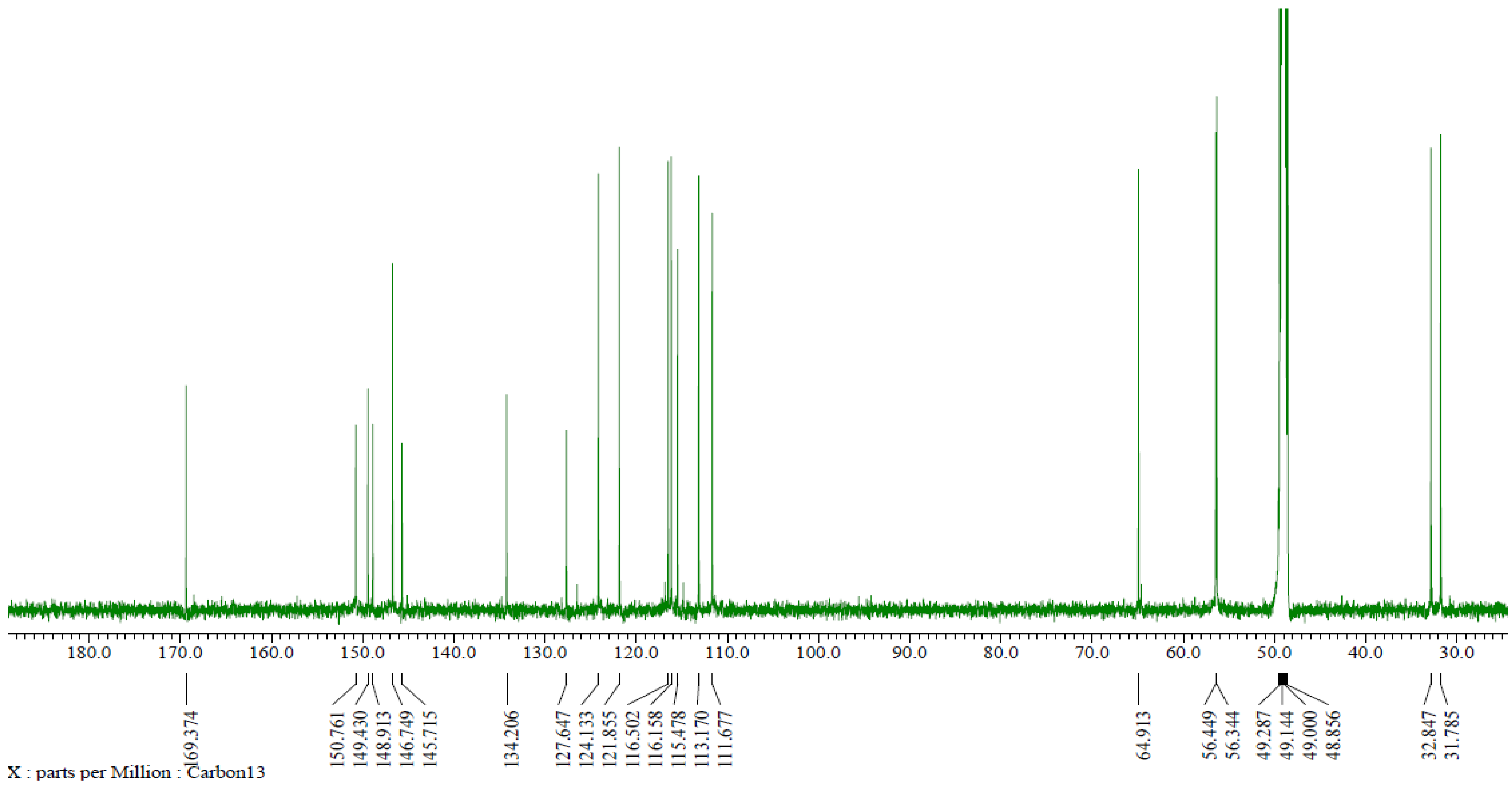

Figure S3. ^{13}C NMR spectrum of the purified compound.

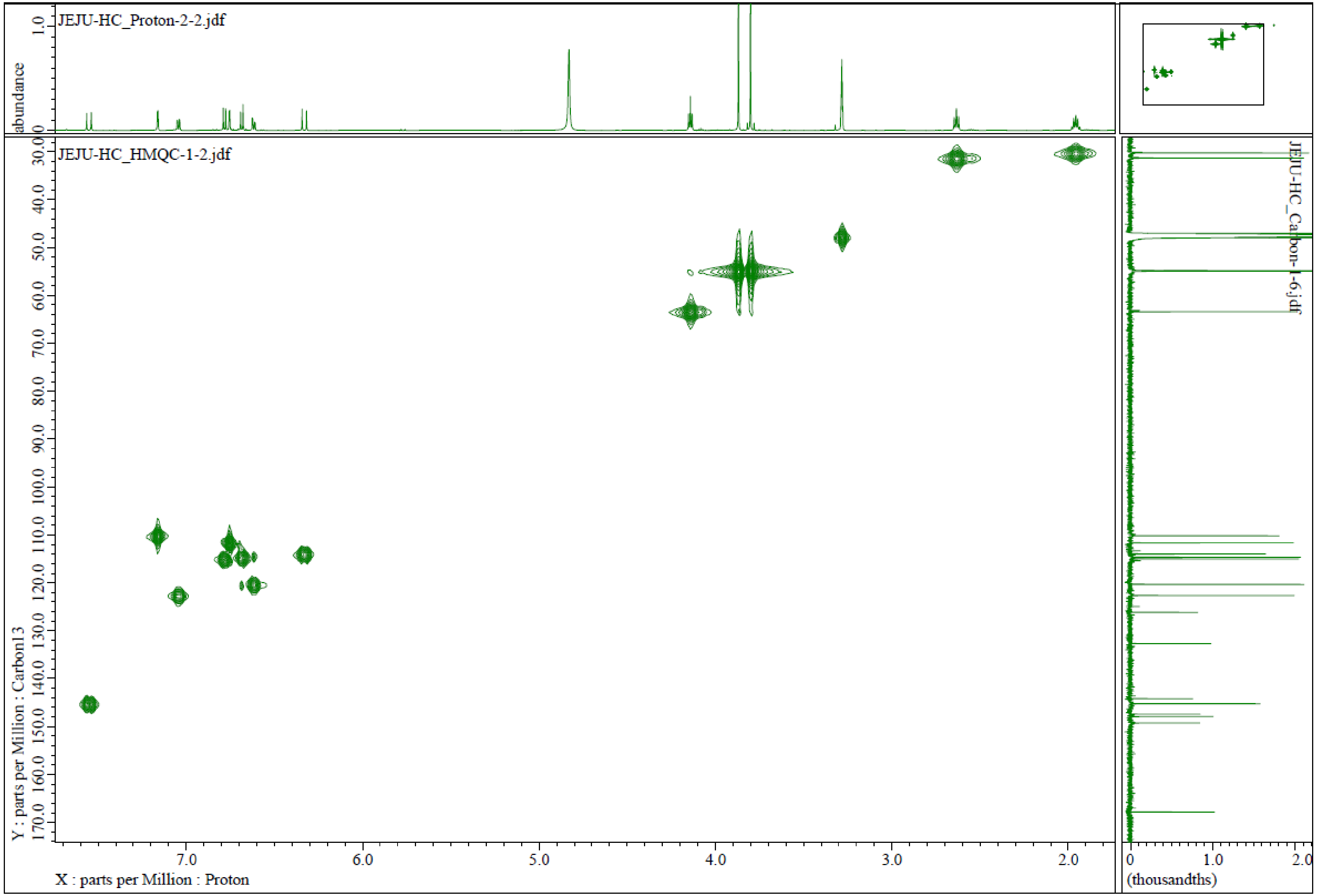

Figure S4. HMQC spectrum of the purified compound.

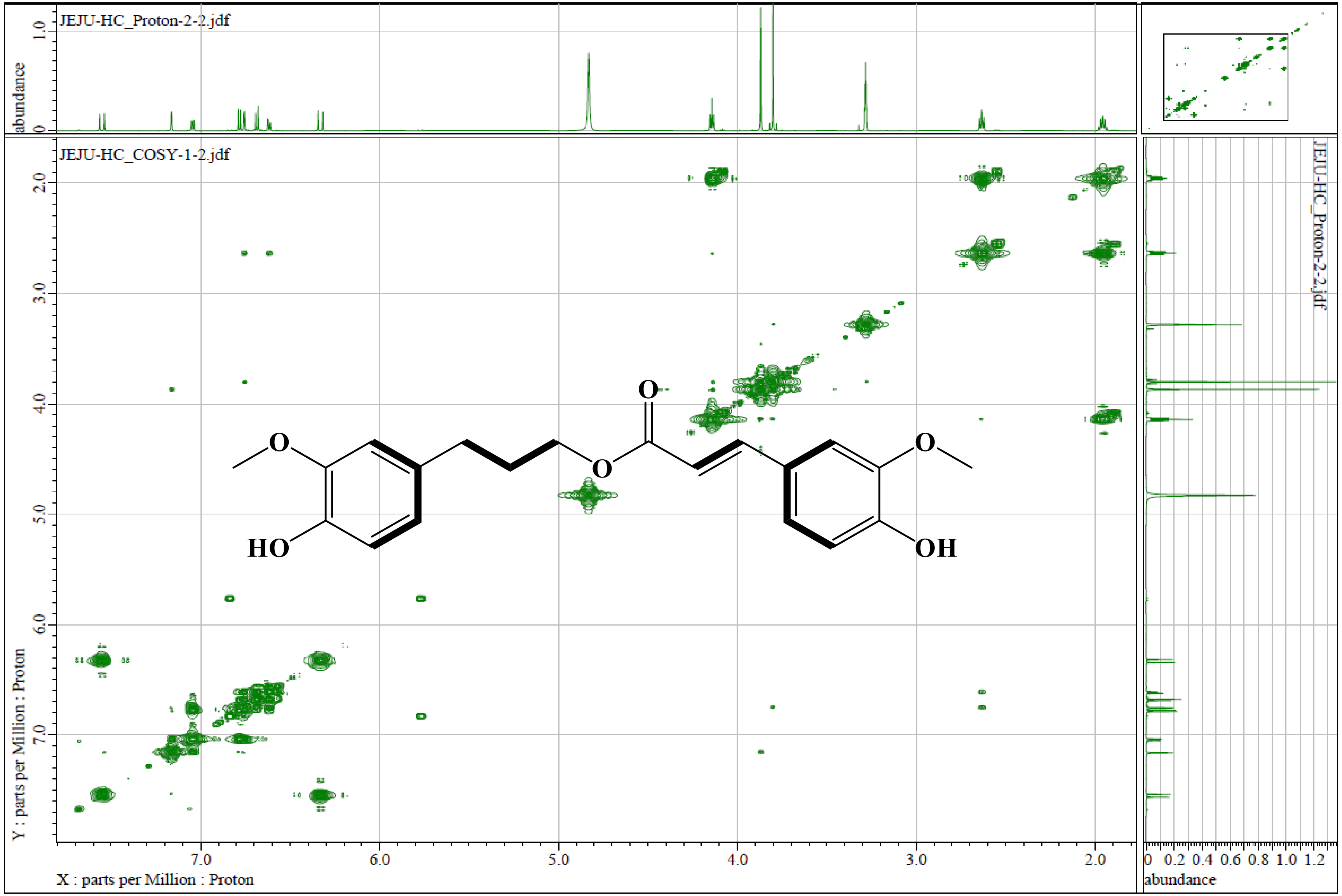

Figure S5. ^{1}H-^{1}H COSY spectrum of the purified compound.

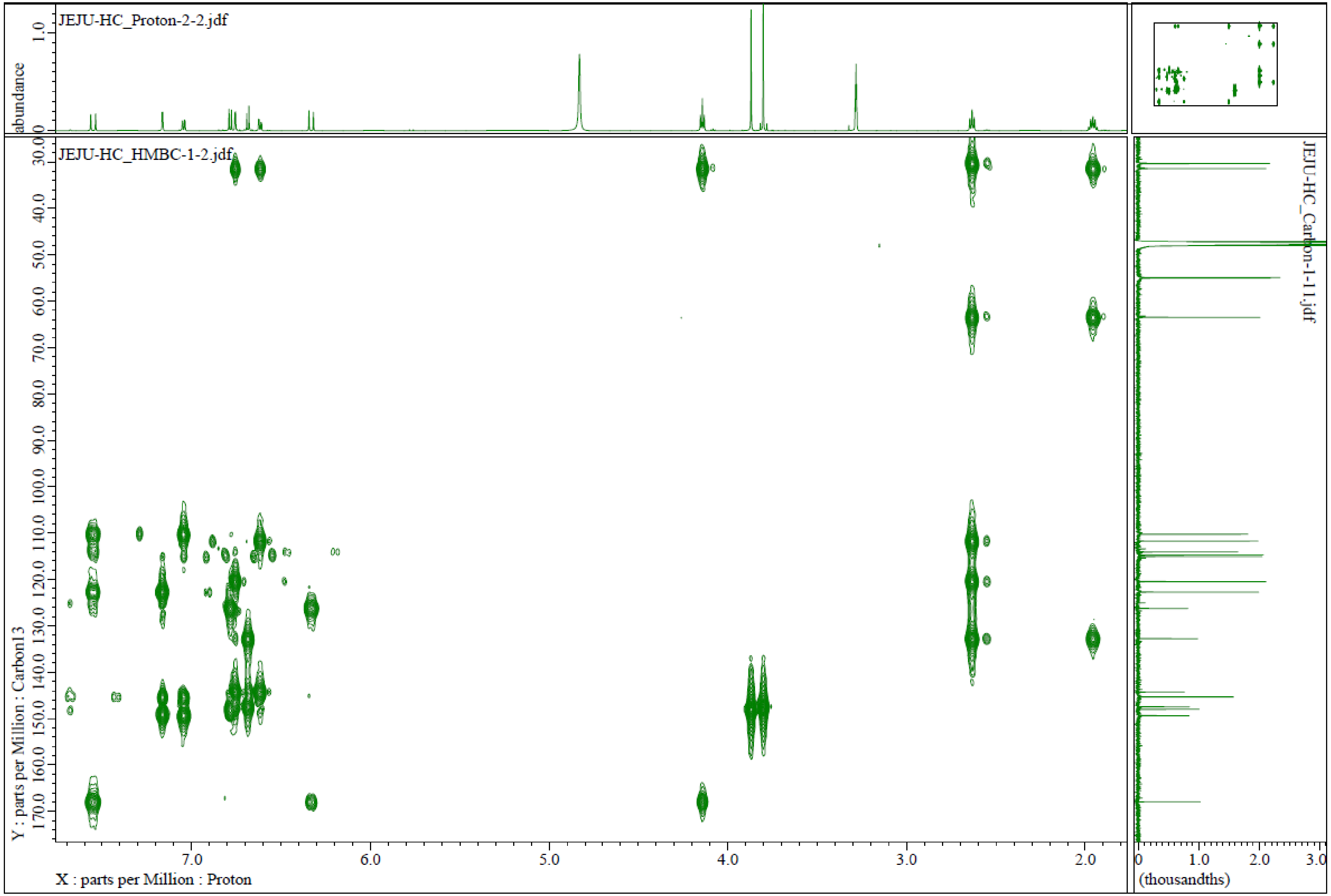

Figure S6. HMBC spectrum of the purified compound.

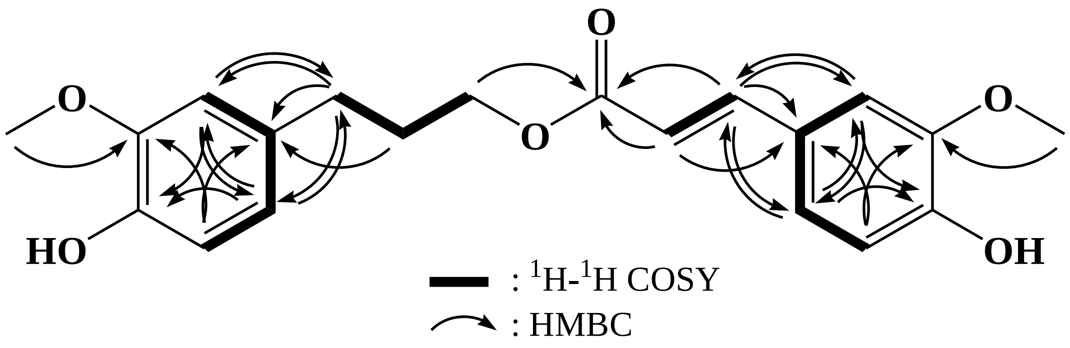

Two-dimensional NMR correlations

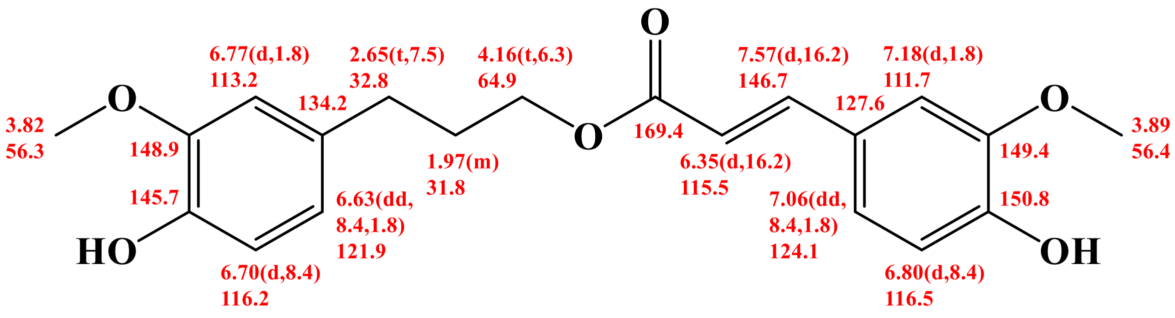

^{1}H and ^{13}C peak assignments

Figure S7. Two-dimensional NMR correlations and ^{1}H, ^{13}C peak assignments of the purified compound.

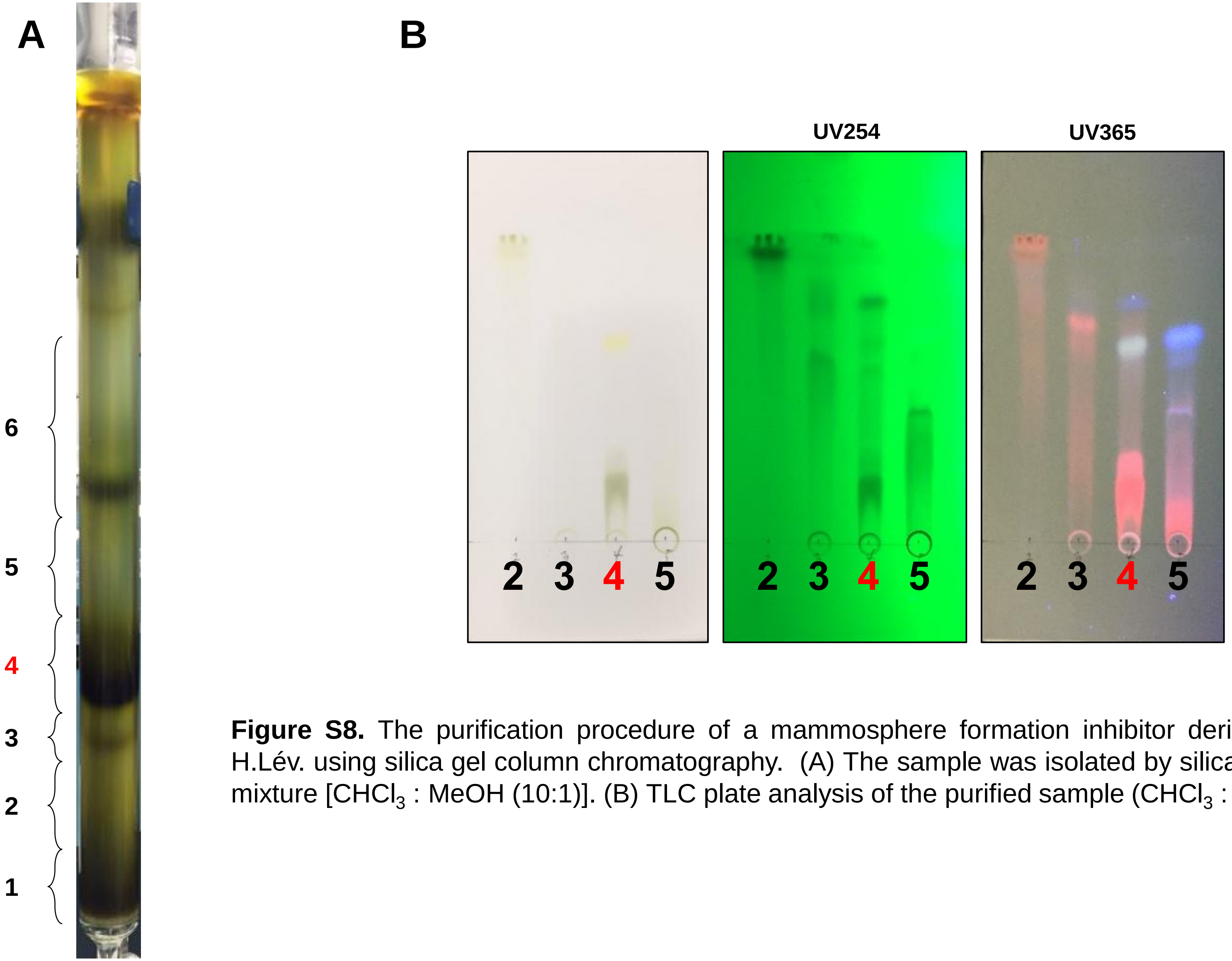

Figure S8. The purification procedure of a mammosphere formation inhibitor derived from *Dendropanax morbiferus* H.Lév. using silica gel column chromatography. (A) The sample was isolated by silica gel chromatography with a solvent mixture [$CHCl_3$: MeOH (10:1)]. (B) TLC plate analysis of the purified sample ($CHCl_3$: MeOH = 10:1). Active fraction: #4.

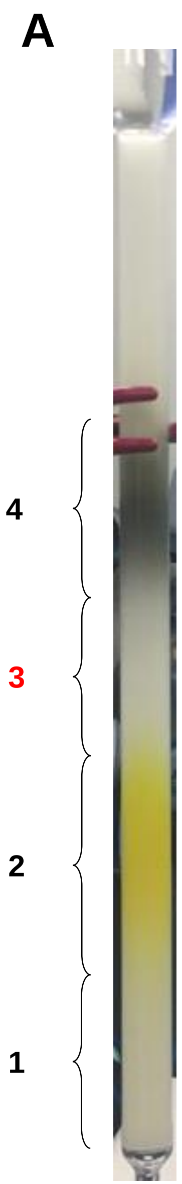

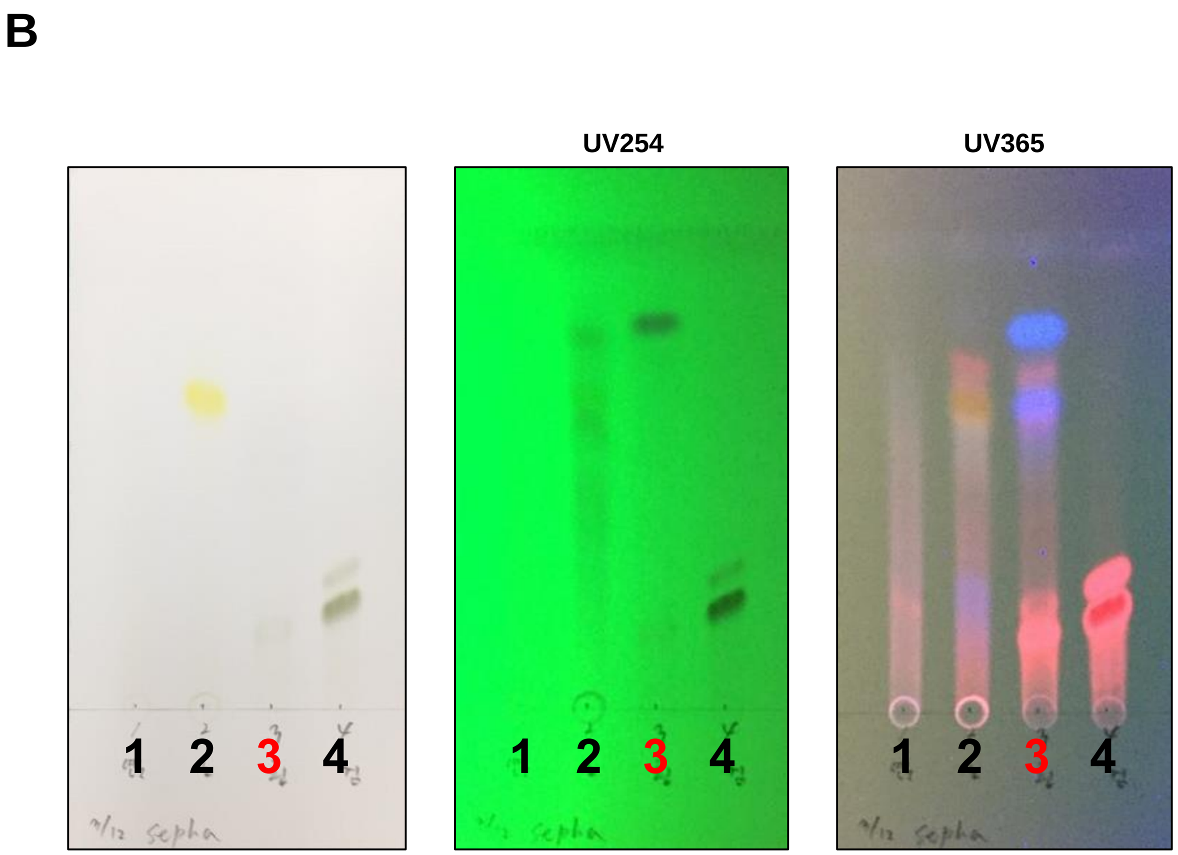

Figure S9. The purification procedure of a mammosphere formation inhibitor derived from *Dendropanax morbiferus* H.Lév. using Sephadex LH-20 column chromatography. (A) The sample was isolated by Sephadex LH-20 column chromatography with MeOH. (B) TLC plate analysis of the purified sample ($CHCl_3$: MeOH = 10:1). Active fraction: #3.

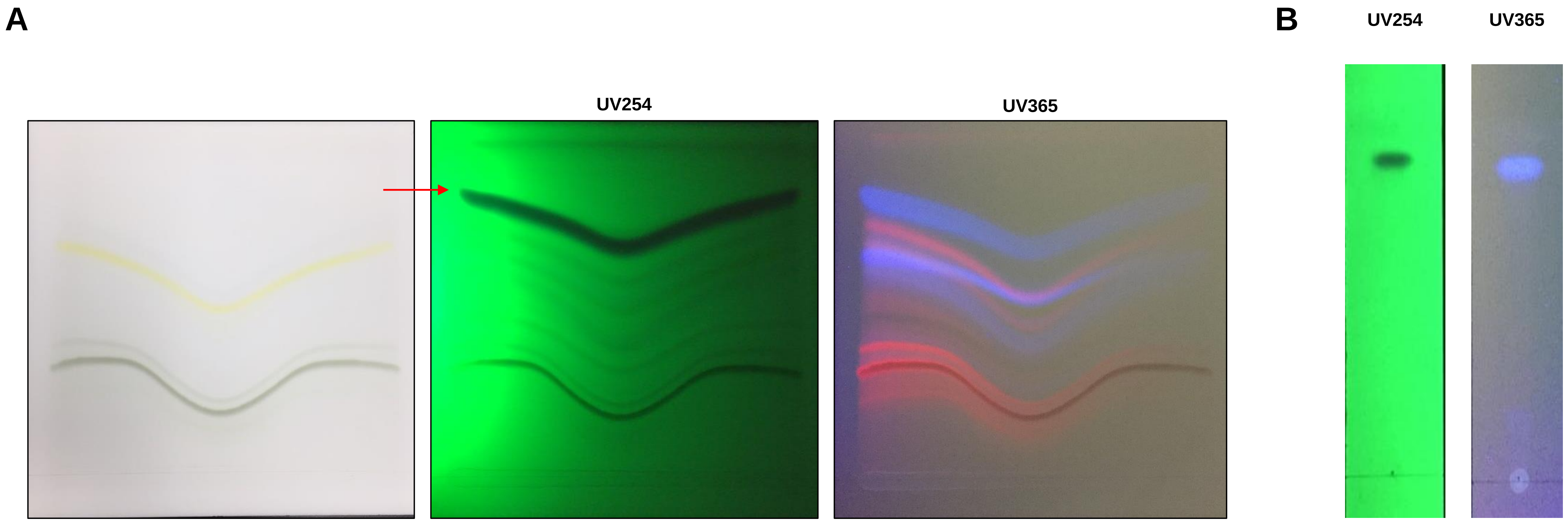

Figure S10. The purification procedure of a mammosphere formation inhibitor derived from *Dendropanax morbiferus* H.Lév. using preparative thin layer chromatography with $CHCl_3$:MeOH (30:1). (A) Preparatory TLC chromatography. (B) TLC plate analysis of the prepared TLC bands after the samples were scraped and purified ($CHCl_3$: MeOH = 10:1).

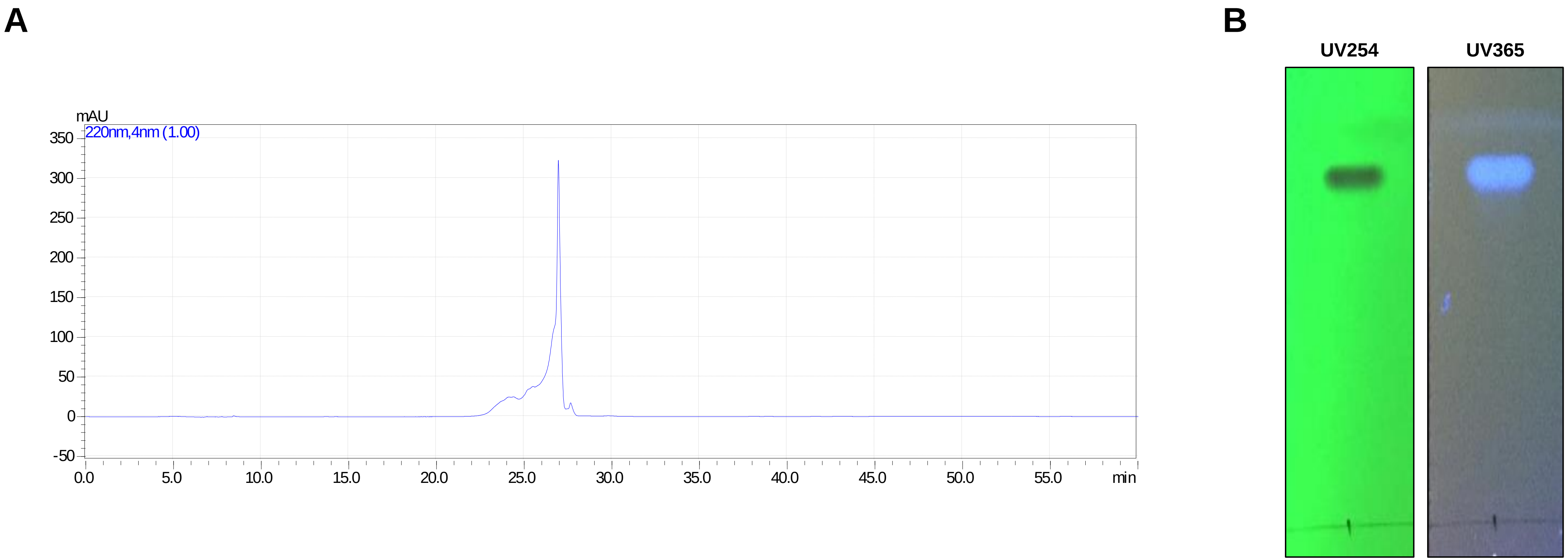

Figure S11. Purification of a mammosphere formation inhibitor derived from *Dendropanax morbiferus* H.Lév. using HPLC. (A) Assessment of the major fraction using HPLC. Samples were collected based on the 220 nm wavelengths. (B) TLC plate analysis of the purified sample ($CHCl_3$: MeOH = 10:1).

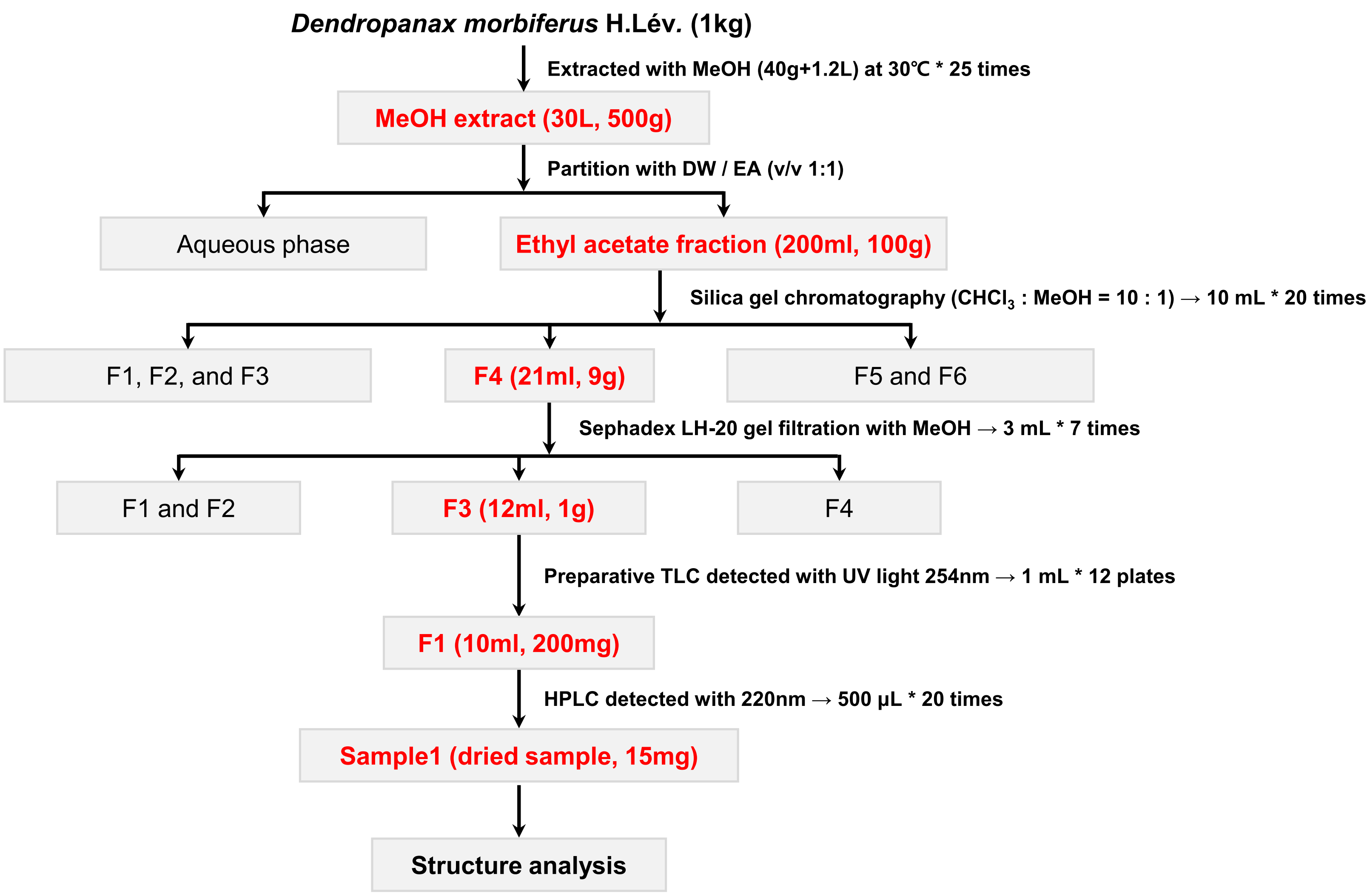

Figure S12. Purification scheme of a mammosphere formation inhibitor derived from *Dendropanax morbiferus* H.Lév.

Table S1. Specific Real-time RT-qPCR primer sequences containing *Nanog, CD44, Oct4, c-myc, Sox2,* and *β-actin* genes

Genes	Primers
Nanog	· Forward: 5′-ATGCCTCACACGGAGACTGT-3′<br>· Reverse: 5′-AAGTGGGTTGTTTGCCTTTG-3′
CD44	· Forward: 5′-AGAAGGTGTGGGCAGAAGAA-3′<br>· Reverse: 5′-AAATGCACCATTTCCTGAGA-3′
Oct4	· Forward: 5′-AGCAAAACCCGGAGGAGT-3′<br>· Reverse: 5′-CCACATCGGCCTGTGTATATC-3′
c-myc	· Forward: 5′-AATGAAAAGGCCCCCAAGGTAGTTATCC-3′<br>· Reverse: 5′-AGCAAAACCCGGAGGAGT-3′
Sox2	· Forward: 5′-TTGCTGCCTCTTTAAGACTAGGA-3′<br>· Reverse: 5′-CTGGGGCTCAAACTTCTCTC-3′
β-actin	· Forward: 5’-TGTTACCAACTGGGACGACA-3’<br>· Reverse: 5’-GGGGTGTTGAAGGTCTCAAA-3
